# Assessment of Cardiac Dysfunction in Patients With Chronic Obstructive Pulmonary Disease (COPD): A Cross-Sectional Study

**DOI:** 10.7759/cureus.39629

**Published:** 2023-05-29

**Authors:** Rehab A Mohammed, Layla A Mohamed, Eman M Abdelsalam, Hend M Maghraby, Nasima M Elkenany, Osama E Nabawi, Intessar Sultan

**Affiliations:** 1 Internal Medicine, Faculty of Medicine for Girls, Al-Azhar University, Cairo, EGY; 2 Internal Medicine, Ibn Sina National College for Medical Studies, Jeddah, SAU; 3 Cardiology, Faculty of Medicine for Girls, Al-Azhar University, Cairo, EGY; 4 Chest Diseases, Al-Azhar University, Cairo, EGY; 5 Internal Medicine, Ibn Sina National College, Jeddah, SAU

**Keywords:** bnp, abg, spirometry, doppler echocardiography, chronic obstructive pulmonary disease

## Abstract

Background: Cardiovascular diseases (CVDs) are frequent in patients having chronic obstructive pulmonary disease (COPD). Despite that, comorbid CVDs receive less guideline-recommended screening in this population compared to others. We aimed to evaluate the cardiac function using echocardiography and to assess spirometry, arterial blood gas (ABG) as well as brain natriuretic peptide (BNP) as prognostic indicators of cardiovascular dysfunction in COPD patients.

Methods: One hundred moderate to very severe COPD patients according to GOLD guidelines with no history of cardiac diseases were recruited from two hospitals in Saudi Arabia and evaluated using electrocardiography (ECG), chest X-ray, BNP, pulmonary functions, ABG analysis, and transthoracic echocardiography. Multiple linear regression analysis was used to determine the predictors of right ventricular (RV) and left ventricular (LV) dysfunction.

Results: Pulmonary hypertension (PH) was detected in 28% of the patients, while 25% had abnormal tricuspid annular plane systolic excursion (TAPSE). Low left ventricular ejection fraction (LVEF) and abnormal LV strain were present in 20%, abnormal right ventricular strain was present in 17%, and abnormal fractional area change (FAC) was detected in 9% of patients. Multiple linear regression analysis was used to explore possible determinants of cardiac function. Age, gender, and the presence of diabetes and hyperlipidemia were significant predictors of cardiac dysfunction in COPD patients. Forced vital capacity (FVC) was an independent predictor of LVEF (odds ratio, OR: 0.424, confidence interval, 95 CI%: 0.025-0.505, p<0.031) and FAC (OR: 0.496, 95 CI%: 0.008-655). Hypoxemia and hypercapnia significantly predict both RV and LV dysfunctions. BNP was an independent predictor of FAC (OR: 0.307, 95 CI%: -0.021, p<0.001).

Conclusion: Cardiac abnormalities are common in moderate to very severe COPD patients. Echocardiography could be considered for the assessment of these patients even in the absence of a history of cardiac disease. Pulmonary functions, ABG, and BNP may offer additional predictive information on cardiac functions in COPD patients.

## Introduction

Chronic obstructive pulmonary disease (COPD) is currently considered as a pulmonary component of systemic endothelial disease in which many cellular and humoral inflammatory processes simultaneously affect multiple organs leading to a state of multi-morbidity [1]. Cardiovascular diseases (CVDs) are one of the most significant co-morbidities in COPD patients with a prevalence ranging from 30% to 60% [2]. Many studies suggest that concomitant CVDs account for 30%-50% of deaths in patients with COPD [3]. COPD affects pulmonary blood vessels, right ventricle, as well as left ventricle leading to the development of pulmonary hypertension (PH), Cor-pulmonale (COR-P), dysrhythmia, and RV and LV dysfunction [4].

Smoking, age, hypoxemia, systemic inflammation, oxidative stress, and increased pulmonary vascular resistance all play a role in this intimate association [1]. From another aspect, shortness of breath and decreased exercise tolerance are present in both diseases; differentiation on a given patient’s symptoms can be challenging and is usually attributed to exacerbation of COPD. So, heart failure in COPD often remains unrecognized [5]. Despite that the current guidelines for the diagnosis and management of COPD emphasize the need for raising awareness of cardiovascular co-morbidities among patients with COPD [6], still, there are no objective recommendations about the assessment and screening tools of heart disease among these individuals. On the other hand, the current global cardiovascular risk estimation models do not include COPD as a risk factor for CVDs in the algorithms, raising the possibility that risk prediction may be sub-optimal [7]. So far, we need to assess the cardiovascular system in COPD patients to identify the complications early and to reduce morbidity and mortality as well [8]. Echocardiography provides a non-invasive, rapid, and reliable screening tool to assess the structural and functional changes of the heart in COPD patients [9]. It is possible that a significant fraction of COPD patients might have clinically silent cardiac abnormalities that can be detected by echocardiography. Therefore, the aim of our study was:

1- To evaluate cardiac functions in COPD patients who had no previous history of cardiac disease using echocardiography. 

2- To assess respiratory functions and brain natriuretic peptide (BNP) as predictors of cardiac abnormalities. 

## Materials and methods

Study design

We conducted this cross-sectional study from January 2022 to January 2023 at two hospitals in Saudi Arabia: Ibn Sina College Hospital in Jeddah and King Khalid Hospital in Hail. The study was performed in compliance with the Helsinki Declaration and in accordance with the regulations approved by the local committee for bioethics research in Hail health affairs via review according to King Abdulaziz City for Science and Technology Good Clinical Practice (KACST GCP) regulations (IRB registration number: H-08-L-074) and (IRB log number: 2021-26). The proposal has undergone a full review by the above-mentioned committee which implements regulations of the law of ethics of research on living creatures in the Kingdom of Saudi Arabia and other applicable national and international regulations.

Study population

A sample size of at least 74 patients was calculated using G*Power 3.1.9.4 with an effect size of 0.15, alpha error probability of 0.05, power of 0.95, degree of freedom (DF) of 57, and the number of predictors set of 16. Therefore, a total of 100 patients were recruited consecutively from inpatient wards of both hospitals who met the criterion for diagnosis of COPD that is based on history, previous physician diagnosis, and presence of irreversible airway obstruction on spirometry in compliance with the latest guidelines for diagnosis and management of COPD [10]. Patients with acute coronary syndrome, documented CVDs, end-stage renal disease, and other respiratory diseases were excluded from the study. The patients are then classified according to the ABCD classification of Global Initiative for Obstructive Lung Disease (GOLD 2020) into GOLD B, C, and D (GOLD A was not included as it includes only mild cases).

Assessments

All patients were subjected to full medical history, clinical examination, plain chest X-ray, electrocardiography (ECG), and spirometry with the following indices recorded; forced expiratory volume in the 1st second (FEV1%), forced vital capacity (FVC%), and FEV1/FVC ratio. Resting ABG analysis on air was done and the following values were recorded: oxygen saturation (SaO2%), partial pressure of oxygen (PaO2 mmHg), partial pressure of carbon dioxide (PaCO2 mmHg), bicarbonate (HCO3 mEq/L), and potential of hydrogen (pH).

Cardiac functions were assessed by resting transthoracic Doppler echocardiography in accordance with the guidelines of the American Society of Echocardiography [10] using Vivid-E9 GE system (GE Ultrasound, Horton, Norway) with tissue Doppler and speckle tracking imaging capability attached to Echo-Pac workstation version (201). The LV and RV functions are assessed using various echo-Doppler modes including M-mode, two-dimensional (2D) echocardiography, conventional Doppler flow imaging, tissue Doppler imaging (TDI), and speckle tracking echocardiography (STE). The following parameters are obtained: 

Right ventricular (RV) parameters included: RV dimensions, tricuspid annular plane systolic excursion (TAPSE), and percentage of FAC. Measures of RV diastolic function by pulsed Doppler through tricuspid flow including tricuspid early and late diastolic filling velocities and tricuspid flow early deceleration time. RVSP was estimated from the tricuspid regurgitant velocity, inferior vena cava diameter, and its collapsibility. The tissue Doppler velocities of the tricuspid valve annulus at the RV free wall are determined. Measured velocities include peak systolic tricuspid annular velocity, early diastolic tricuspid annular velocity, and late diastolic tricuspid annular velocity and RV global longitudinal strain (GLS) by 2D speckle tracking. 

Left ventricular (LV) parameters included: LV internal dimensions and M-mode/2D echo ejection fraction (EF). Doppler Echocardiography was used to measure mitral inflow velocity including early, late diastolic velocities and deceleration time. Tissue Doppler velocities were obtained by calculating the average of early and late diastolic velocities from four mitral annuli. LV systolic function was measured by 2D speckled tracking echo.

Diagnosis of abnormality

An elevated RV/pulmonary artery systolic pressure (PASP) >35 mmHg indicated PH. TAPSE is known as a noninvasive surrogate for RVEF. It is considered as an accurate measure of RV function in PH patients, a TAPSE value < 17 mm indicates RV systolic dysfunction. FAC provides an estimate of the global RV systolic function, values <35% indicate systolic dysfunction. Right ventricular (RV)-global longitudinal strain (RV-GLS) is a well-established tool to detect early RV dysfunction, a value of <20% suggested RV dysfunction. LV-global longitudinal strain (LV-GLS) is a well-established tool to detect early LV dysfunction, a value of <17%, indicated LV systolic dysfunction. Left ventricular ejection fraction (LVEF) is the central measure of LV systolic function, a value of <50% indicates LV systolic dysfunction [11].

Statistical analysis

Data were analyzed using the SPSS software package, version 20.0 (SPSS Inc., Chicago, IL, USA). The normality was tested using the Shapiro-Wilk test. Normally distributed data are presented as mean ± standard deviation (SD). Non-normally distributed variables are expressed as the median and interquartile range (25th-75th percentile). Categorical variables are presented as numbers and percentages. Multiple linear regression analysis was used to determine factors related to cardiac dysfunction and to assess the effect of selected variables on ventricular systolic and diastolic function. Standardized coefficient beta (odd ratio) was interpreted as the absolute change in dependent variables per change in cardiac function. A p-value <0.05 was considered statistically significant. 

## Results

Baseline characteristics

**Figure 1:**
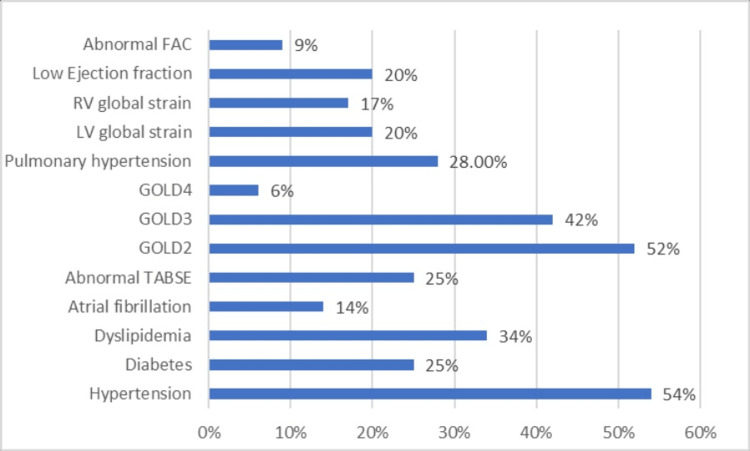
Baseline characteristics of COPD patients. COPD, chronic obstructive pulmonary disease; FAC, fractional area change; TAPSE, tricuspid annular plane systolic excursion

The study included 86 males and 14 females. Their mean age was 67.09 ± 6.38 years, ranging from 54 to 79 years. Most patients (52%) had moderate airflow limitation (GOLD 2); 42% had severe airflow limitation (GOLD 3), and only 6% of them had very severe airflow limitation (GOLD 4) (Figure 1).

Hypertension was the most associated comorbidity (54%) followed by dyslipidemia (34%) and diabetes (25%). Atrial fibrillation (AF) was detected in 14% of patients. All the pulmonary function parameters were below normal limits (Table 1), where the FEV1% predicted was 51.77 ± 11.53 and the FVC% was 54.73 ± 10.59. Their mean partial pressure of oxygen (PaO2) was 55.61 ± 5.96 mmHg, partial pressure of carbon dioxide (PaCO2) was 54.37 ± 10.58 mmHg, and oxygen saturation was 86.88% ± 4.93% reflecting type 2 respiratory failure (Table 1). 

**Table 1:** Spirometric pulmonary functions and ABG parameters of the studied sample. FVC, forced vital capacity; FEV1, forced expiratory volume 1; pH, potential of hydrogen; PaO2, partial pressure of oxygen; PaCO2, partial pressure of carbon dioxide; HCO3, bicarbonate; ABG, arterial blood gas; SD, standard deviation

Variables	All cases (n = 100) Mean ± SD
FVC (%)	54.73 ± 10.59
FEV1/FVC	51.12 ± 10.92
FEV1 (%)	51.77 ± 11.53
Reversibility (%)	6 (1-10)
pH	7.33 ± 0.03
PaO_2 _(mmHg)	55.61 ± 5.96
PaCO_2 _(mmHg)	54.37 ± 10.58
Oxygen saturation (%)	86.88 ± 4.93
HCO_3 _(mEg/L)	31.08 ± 5.28

Echocardiographic parameters were all within the normal range (Table 2).

**Table 2:** Echocardiographic and BNP parameters of the studied sample. LA, left atrium; LV EDD, left ventricular end diastolic diameter; LV ESD, left ventricular end systolic diameter; IVSTD, interventricular septal thickness at end diastole; PWTD, posterior wall thickness at end diastole; FS, fractional of shortening; EF mode, ejection fraction by M-mode; EF 2D, ejection fraction by 2-dimensional; RVOR-PD, right ventricular outflow tract - proximal diameter; RVOT-DD, right ventricular outflow tract - distal diameter; FAC, fractional area change; RV basal, right ventricular basal diameter; RV mid, right ventricular mid diameter; RV long, right ventricular longitudinal diameter; TAPSE, tricuspid annular plane systolic excursion; A/E, early to Atrial filling velocity; PV/ET, pulmonary valve to ejection time ratio; RVGS, right ventricular global longitudinal strain; RVSP, right ventricular systolic pressure; LVGS, left ventricular global strain; BNP, brain natriuretic peptide

Variables	All cases (n = 100) Mean ± SD
LA (mm)	35.37 ± 5.02
LV EDD (mm)	45.6 ± 4.44
LV ESD (mm)	29.93 ± 5.37
IVSTD (mm)	9.45 ± 1.72
PWTD (mm)	9.14 ± 1.79
FS	37.73 ± 3.63
EF (%) mode	67.45 ± 4.77
EF (%) 2D	56.87 ± 7.25
RVOT-PD (mm)	30.32 ± 3.7
RVOT-DD (mm)	21.25 ± 3.39
FAC (%)	46.97 ± 8.57
RV. basal (mm)	33.32 ± 6.87
RV. mid (mm)	28.08 ± 6.96
RV. long (mm)	62.36 ± 10.38
TAPSE (mm)	20.48 ± 3.48
A/E (mm)	374.04 ± 52.91
PV/ET (mm)	286.0 ± 36.26
Tei index	0.334 ± 0.12
RVGS (mm)	20.8 ()
RVSP (mm)	29.0 ()
LVGS (mm)	19.1 ()
BNP (pg/mm) median (min-max)	187 (74-769)

However, when applying the cut-off criteria for abnormality; 28% of patients had high PASP (PH), and 20% had low LVEF. Abnormal LV global strain was detected in 20% and RV global strain was detected in 17%. Abnormal TAPSE was detected in 25% of patients while 9% had low FAC (Figure 1).

Multivariate regression analysis was used to analyze the determinants of cardiovascular risk in COPD patients. The model was based on multiple variables including the demographic characteristics, the presence of comorbidities, the spirometry indices, the ABG parameters, and the cardiac troponin. Within the model, age (odd ratio, OR: 0.270, confidence interval, 95 CI%: 0.077-0.639), BNP (OR: -0.307, 95 CI%: -0.021 - -0.001), and FVC (OR: 0.496, 95 CI%: 0.008-655) were the significant predictors of FAC (Table 3).

**Table 3:** Predictors of FAC. CI, confidence interval; DM, diabetes mellitus; AF, atrial fibrillation; BNP, brain natriuretic peptide; FVC, forced vital capacity; GOLD, global initiative for obstructive lung disease; FEV1, forced expiratory volume 1; pH, potential of hydrogen; PaO2, partial pressure of oxygen; PaCO2, partial pressure of carbon dioxide; HCO3, bicarbonate; FAC, fractional area change

	Standard coefficients beta	p	95.0% CI	
			Lower bound	Upper bound
(Constant)		0.084	-48.780	763.302
Age	0.270	0.013	0.077	0.639
Gender	0.023	0.828	-3.475	4.326
Hypertension	0.122	0.241	-1.476	5.776
DM	-0.101	0.377	-6.191	2.373
Hyperlipidemia	0.130	0.250	-1.654	6.241
Heart rate	-0.072	0.500	-0.182	0.090
AF	0.109	0.319	-2.297	6.957
BNP	-0.307	0.028	-0.021	-0.001
FVC	0.496	0.008	0.105	0.655
GOLD Severity	-0.044	0.835	-7.406	6.000
FEV1/FVC	-0.043	0.693	-0.215	0.143
FEV1	0.015	0.958	-0.396	0.418
Ph	-0.177	0.102	-100.878	9.267
PaO_2_	0.028	0.814	-0.295	0.373
PaCO_2_	-0.037	0.747	-0.218	0.157
HCO_3_	-0.150	0.164	-0.576	0.100

While PaO2 was the significant predictor of RV-GLS (OR: 0.310, 95% CI: 0.047-0.421) (Table 4),

**Table 4:** Predictors of RV global strain. CI, confidence interval; DM, diabetes mellitus; AF, atrial fibrillation; BNP, brain natriuretic peptide; FVC, forced vital capacity; GOLD, global initiative for obstructive lung disease; FEV1, forced expiratory volume 1; pH, potential of hydrogen; PaO2, partial pressure of oxygen; PaCO2, partial pressure of carbon dioxide; HCO3, bicarbonate

	Standard coefficients beta	p	95.0% CI	
			Lower bound	Upper bound
(Constant)		0.206	-82.043	373.065
Age	-0.009	0.933	-0.164	0.151
Gender	-0.172	0.122	-3.900	0.472
Hypertension	-0.044	0.688	-2.443	1.621
DM	-0.097	0.423	-3.368	1.431
Hyperlipidemia	-0.170	0.156	-3.802	0.623
Heart rate	-0.024	0.831	-0.068	0.084
AF	-0.059	0.611	-3.257	1.929
BNP	0.245	0.096	-0.001	0.010
FVC	0.216	0.260	-0.066	0.242
GOLD Severity	0.065	0.773	-3.211	4.302
FEV/FVC	0.185	0.112	-0.019	0.181
FEV1	-0.040	0.895	-0.243	0.213
pH	-0.125	0.272	-47.975	13.753
PaO_2_	0.310	0.015	0.047	0.421
PaCO_2_	0.152	0.206	-0.038	0.172
HCO_3_	0.027	0.814	-0.212	0.167

RVSP (OR - 0.346, -1.495 - -0.263, p<0.006) (Table 5),

**Table 5:** Predictors of LV global strain. CI, confidence interval; DM, diabetes mellitus; AF, atrial fibrillation; BNP, brain natriuretic peptide; FVC, forced vital capacity; GOLD, global initiative for obstructive lung disease; FEV1, forced expiratory volume 1; pH, potential of hydrogen; PaO2, partial pressure of oxygen; PaCO2, partial pressure of carbon dioxide; HCO3, bicarbonate

	Standard coefficients beta	p	95.0% CI	
			Lower bound	Upper bound
Age	-0.127	0.274	-0.189	0.054
Gender	-0.250	0.030	-3.562	-0.186
Hypertension	-0.116	0.305	-2.382	0.756
DM	-0.127	0.308	-2.806	0.900
Hyperlipidemia	0.009	0.942	-1.645	1.771
Heart rate	-0.057	0.621	-0.073	0.044
AF	0.016	0.894	-1.868	2.137
BNP	0.269	0.076	-0.000	0.008
FVC	0.251	0.204	-0.042	0.196
GOLD severity	-0.34	0.883	-3.115	2.687
FEV/FVC	0.166	0.164	-0.023	0.132
FEV1	-0.028	0.927	-0.184	0.168
pH	-0.080	0.493	-32.057	15.613
PaO_2_	0.302	0.021	0.027	0.316
PaCO_2_	-0.135	0.276	-0.126	0.37
Oxygen saturation	-0.141	0.222	-0.267	0.063
HCO_3_	-0.019	0.869	-0.158	0.134

and LV-GLS (OR: 0.021, 95 CI%: 0.027 - 0.316, p<0.021) (Table 6). 

**Table 6:** Predictors of RV systolic pressure. CI, confidence interval; DM, diabetes mellitus; AF, atrial fibrillation; BNP, brain natriuretic peptide; FVC, forced vital capacity; GOLD, global initiative for obstructive lung disease; FEV1, forced expiratory volume 1; pH, potential of hydrogen; PaO2, partial pressure of oxygen; PaCO2, partial pressure of carbon dioxide; HCO3, bicarbonate

	Standard coefficients beta	p	95.0% CI	
			Lower bound	Upper bound
(Constant)		0.913	-707.642	789.798
Age	-0.009	0.934	-0.539	0.496
Gender	0.135	0.213	-2.663	11.721
Hypertension	-0.081	0.454	-9.211	4.161
DM	-0.002	0.988	-7.955	7.836
Hyperlipidemia	0.222	0.060	-0.314	14.244
Heart rate	0.236	0.034	0.021	0.521
AF	0.083	0.0464	-5.381	11.683
BNP	0.189	0.187	-0.006	0.030
FVC	-0.209	0.266	-0.792	0.222
GOLD Severity	0.040	0.857	-11.242	13.478
FEV/FVC	-0.078	0.488	-0.446	0.215
FEV1	-0.078	0.791	-0.851	0.650
pH	-0.002	0.985	-102.479	100.622
PaO_2_	-0.346	0.006	-1.495	-0.263
PaCO_2_	0.054	0.645	-0.265	0.426
HCO_3_	-0.089	0.422	-0.875	0.371

Male gender predicted LV-GLS (OR: -0.250, 95 CI%: -3.562 - -0.186, p<0.030) and EF (OR: 0.253, 95 CI%: -7.288 - -0.486, p<0.026) (Table 7).

**Table 7:** Predictors of ejection fraction by 2-dimensional. CI, confidence interval; DM, diabetes mellitus; AF, atrial fibrillation; BNP, brain natriuretic peptide; FVC, forced vital capacity; GOLD, global initiative for obstructive lung disease; FEV1, forced expiratory volume 1; pH, potential of hydrogen; PaO2, partial pressure of oxygen; PaCO2, partial pressure of carbon dioxide; HCO3, bicarbonate; EF 2D, ejection fraction by 2-dimensional

	Standard coefficients beta	p	95.0% CI	
			Lower bound	Upper bound
Age	-0.047	0.681	-0.295	0.194
Gender	-0.253	0.026	-7.288	-0.486
Hypertension	-0.025	0.824	-3.515	2.809
DM	-0.121	0.324	-5.591	1.876
Hyperlipidemia	0.117	0.333	-1.762	5.123
Heart rate	0.140	0.220	-0.45	0.192
AF	0.014	0.908	-3.799	4.271
BNP	0.178	0.228	-0.003	0.014
FVC	0.424	0.031	0.025	0.505
GOLD severity	-0.081	0.723	-6.887	4.804
FEV1/FVC	-0.032	0.780	-0.178	0.134
FEV1	-0.240	0.431	-0.496	0.214
pH	-0.145	0.206	-78.719	17.334
PaO_2_	0.197	0.121	-0.062	0.520
PaCO_2_	-0.124	0.305	-0.248	0.079
Oxygen saturation	-0.077	0.493	-0.447	0.217
HCO_3_	0.084	0.466	-0.187	0.403

Moreover, FVC was the significant predictor of LVEF (OR: 0.424, 95 CI%: 0.025 - 0.505, p<0.031) (Table 7). Significant predictors for TAPSE were the presence of diabetes (OR: -0.224, 95% CI: -3.6- -0.026, p<0.047), and arterial PaCO2 (OR: 0.338, 95% CI: 0.040-0.203, p<0.004) (Table 8). The degree of airway obstruction as determined by GOLD is not related to cardiac dysfunction across all analyzed models (Tables 3-8).

**Table 8:** Predictors of TAPSE. CI, confidence interval; DM, diabetes mellitus; AF, atrial fibrillation; BNP, brain natriuretic peptide; FVC, forced vital capacity; FEV1, forced expiratory volume 1; pH, potential of hydrogen; PaO2, partial pressure of oxygen; PaCO2, partial pressure of carbon dioxide; HCO3, bicarbonate; TAPSE, tricuspid annular plane systolic excursion

	Standard coefficients beta	p	95.0% CI	
			Lower bound	Upper bound
Age	0.042	0.685	-0.095	0.144
Gender	-0.106	0.336	-2.654	0.919
Hypertension	0.128	0.213	-0.574	2.528
DM	-0.224	0.047	-3.602	-0.026
Hyperlipidemia	0.007	0.951	-1.695	1.803
AF	0.034	0.754	-1.682	2.311
BNP	0.094	0.516	-0.003	0.006
FVC	0.143	0.504	-0.091	0.184
FEV1/FVC	0.182	0.086	-0.010	0.143
FEV1	-0.028	0.897	-0.141	0.123
pH	-0.126	0.252	-2.924	0.778
PaO_2_	0.235	0.053	-0.002	0.341
PaCO_2_	0.338	0.004	0.040	0.203
Oxygen saturation	-0.135	0.225	-0.274	0.066
HCO_3_	-0.041	0.693	-0.170	0.113

## Discussion

In this study, the most common echocardiographic abnormalities were PH (28%) followed by TAPSE (25%). Many studies demonstrated a reasonable correlation between pulmonary arterial pressure measured by echocardiography and pressure measured by right heart catheterization [11]. The European Society of Cardiology/European Respiratory Society (2015) recommended echocardiography as a non-invasive test for PH screening and follow-up [12]. PH in COPD can be caused by many factors including hypoxia, chronic hypercapnia, and metabolic alkalosis [13].

For a long time, the evaluation of RVSP in COPD patients was the main interest of both pulmonologists and cardiologists [14]. In this study, we attempted to go beyond RVSP and more recent echocardiographic techniques such as RV strain by speckle tracking echocardiography (STE) were used. We found that 17% of COPD patients have impaired RV strain and 9% of cases had abnormal FAC. In contrast to our results, Nasir et al. [14] found that more than 50% of COPD patients have RV dysfunction when based on RV basal strain compared to 25% and 30% based on TAPSE and FAC.

Even though cardiac abnormalities in COPD have often been mainly assessed with respect to the right ventricle [14-15], the present study showed that 20% of patients had impaired LV function as measured by LV strain analysis and LVEF. It has been demonstrated that the measurement of LVGLS enhances the risk stratification of heart failure and identifies those who have myocardial decompensation despite normal LVEF. Therefore, it can provide additional prognostic information which could be missed when EF is only measured [16]. According to Macchia et al. [16] results, systolic dysfunction was found in 14% of COPD patients, and throughout a 2-year follow-up, patients with COPD accompanying ventricular dysfunction had an adjusted mortality risk two times higher than that of patients without ventricular dysfunction.

In this study, we used a multivariate model to investigate possible predictors of cardiac function in COPD patients. The results revealed that age, male gender, and the presence of diabetes and hyperlipidemia were predictors of cardiac dysfunction in our patients. These findings are in line with studies conducted by Feary et al. [17] in the UK which demonstrated that COPD patients in their late-to-middle ages have the highest risk of developing cardiovascular disease. Recent studies suggest that lipids can contribute not only to the pathogenesis of CVD but also to COPD. Oxidized low-density lipoprotein stimulates the production of reactive oxygen species (ROS) and pro-inflammatory cytokines that have a role in atherogenesis as well as COPD pathogenesis [18]. Moreover, studies suggest that increased apoptosis of pulmonary epithelial cells is enhanced by dysregulation of lipid metabolism [19].

Pulmonary function indices were tested as a possible determinant of cardiac function among our patients. Our results revealed that FVC was the only independent predictor of cardiac function and according to our findings, FVC was associated with FAC and LVEF. In a study done on 57 Duchene muscular dystrophy patients, right ventricular ejection fraction (RVEF) and right ventricular end diastolic volume (RVEDVI) were related to FVC% which suggests that worsening respiratory function may have a negative impact on the heart and can be used to monitor the cardiac function in these patients [20]. Das et al. [21] found that FAC of the right ventricle was positively correlated with FEV1 and FEV1/ FVC ratio. Baum et al. [22] concluded that FVC, FEV1, and their ratio (FEV1/FVC) were associated with LV systolic and diastolic function. In contrast, neither FVC nor FEV1 was related to cardiovascular risk in COPD patients in a study conducted by Bikov et al. [23]. 

In our study, hypoxemia was the persistent significant predictor of many cardiac parameters including RVSP, RVGLS, and LVGS. Hypoxia is associated with increased systemic inflammation, oxidative stress, and endothelial dysfunction which may all contribute to cardiovascular disease [24]. Wells et al. found that hypoxemia over a 5-year period is associated with comorbid heart failure, pulmonary artery enlargement, and severe COPD exacerbation. They advised to consider blood gas parameters as detrimental prognostic factors in right-side heart failure [25]. TAPSE is known as a noninvasive surrogate for RVEF, and the degree of the RV function [26]. In our study, we found that PaCO2 significantly predicts the TAPSE. This is in accordance with Terzano et al. [27] who reported a significant correlation between ABG and TAPSE in COPD patients and this relation was independent of the severity of COPD. These findings further demonstrate the relationship between COPD and RV function and support the usage of blood gas parameters as a prognostic factor of cardiac functions in COPD.

Given the recognized cardiovascular phenotypes within COPD, the use of validated cardiovascular biomarkers is warranted [28]. Natriuretic peptides (NPs) are validated markers of impaired myocardial function, and they are routinely used in the stratification of acute and chronic HF patients [29]. However, the prognostic role of BNP in COPD patients remains unclear. In this study, we aimed to assess the levels of BNP and their association with cardiac dysfunction in patients with COPD. The BNP levels were normal in COPD patients; however, it significantly predicts FAC and RV strain in this population. A study by Sharif et al. [30] demonstrated that BNP levels were correlated with RV remodeling as well as the severity of PH. Arief et al. [31] demonstrated a significant correlation between BNP and abnormal RV findings on echocardiography including RV dilatation and RV dysfunction. Tian et al. [32] suggest that BNP might have a strong diagnostic efficacy to predict morbidity in patients with COPD.

The present study had some limitations: as it was a cross-sectional study, the absolute causal relationship between COPD and cardiac dysfunction cannot be confirmed. Baseline comorbidities were reported by patients themselves and not verified by detailed laboratory tests, which may lead to recall bias. The PASP was estimated by echocardiographic Doppler study only and not confirmed by RHC, which is the gold standard method to assess PH. Although we excluded patients with a history of CAD, the presence of CAD among included patients was not excluded by interventional angiography. Additionally, the absence of data about COPD treatment and its effect on echocardiographic parameters is another limitation of this study.

## Conclusions

Cardiovascular diseases are common among patients with moderate-to-very-severe COPD. Worsening respiratory functions may have a negative impact on the heart and can be used to monitor the cardiac function in these patients. In addition to the standard cardiac risk factors, FVC, hypoxia, and BNP may add predictive information about cardiac functions in COPD patients. Echocardiography screening and evaluation of both RV and LV functions in COPD patients can identify patients who need close monitoring and intense treatment, thus improving their prognosis.
